# Impact of Varying Resuscitation Proportions of Resuscitated Patients on VA-ECMO Outcomes in the ECLS-SHOCK Trial

**DOI:** 10.1016/j.jacadv.2026.102585

**Published:** 2026-01-29

**Authors:** Tharusan Thevathasan, Uwe Zeymer, Anne Freund, Janine Pöss, Steffen Schneider, Holger Thiele, Steffen Desch

**Affiliations:** a DZHK (German Center for Cardiovascular Research), Germany; b Department of Cardiology, Angiology and Intensive Care Medicine, Deutsches Herzzentrum der Charité (DHZC), Berlin, Germany; c Berlin Institute of Health, Berlin, Germany; d Institut für Herzinfarktforschung, Ludwigshafen, Germany; e Department of Cardiology and Angiology, University Heart Center Freiburg-Bad Krozingen, Faculty of Medicine, University of Freiburg, Freiburg, Germany; f Department of Internal Medicine/Cardiology, Heart Center Leipzig at Leipzig University, Leipzig, Germany

**Keywords:** acute myocardial infarction, cardiogenic shock, cardio-pulmonary resuscitation, mechanical circulatory support

**What is the clinical question being addressed?**

Does the proportion of patients with preceding cardiopulmonary resuscitation influence the effect of venoarterial extracorporeal membrane oxygenation on 30-day and 1-year mortality in the ECLS-SHOCK trial?

**What is the main finding?**

Across analytic and resampling approaches, venoarterial extracorporeal membrane oxygenation’s treatment effects remained neutral irrespective of cardiopulmonary resuscitation proportion.

The ECLS-SHOCK (Extracorporeal Life Support Infarct-Related Cardiogenic Shock) trial showed no survival benefit of venoarterial extracorporeal membrane oxygenation (VA-ECMO) in patients with acute myocardial infarction complicated by cardiogenic shock.^1^^,^^2^ A major point of debate has been that the high proportion of patients with preceding cardiopulmonary resuscitation (CPR) (77%) may have diluted a potential VA-ECMO effect. In contrast, the DanGer Shock (Danish-German Cardiogenic Shock) trial, which excluded comatose CPR patients, demonstrated a mortality benefit with microaxial flow pumps.^3^ A prespecified subanalysis of the ECLS-SHOCK trial compared outcomes between patients with and without CPR, and found no significant interaction between the resuscitation status and VA-ECMO treatment effect.^4^ Building on the prior analysis, this study extends the evidence by applying analytic mixture and bootstrap resampling approaches to simulate trial populations with varying CPR proportions, thereby directly testing whether case-mix differences could plausibly account for the neutral overall result.

## Methods

Data from the ECLS-SHOCK trial were used, with 30-day and 1-year all-cause mortality as the primary outcomes. Details on design and results of the trial were published previously.^1^ The study was conducted according to the Declaration of Helsinki and approved by the local ethics committees (name: “Ethikkommission”; IRB approval date: January 14, 2019; approval number: 267/18-ek; study title: ECLS-SHOCK) and all patients or their legal representatives gave written informed consent. First, the treatment effect of VA-ECMO vs control was estimated separately for patients with and without preceding CPR using logistic regression, yielding subgroup-specific ORs. These subgroup estimates served as the basis for subsequent analyses.

In the analytic mixture approach, treatment effects of VA-ECMO were first estimated separately in resuscitated and nonresuscitated patients using logistic regression, yielding subgroup-specific ORs with corresponding CIs; these estimates were then mathematically combined in varying proportions to generate hypothetical trial populations with CPR prevalence ranging from 0% to 100% in 10% increments, allowing assessment of how the overall treatment effect would change with different case-mix compositions. In the resampling approach, synthetic trial cohorts were constructed for each target CPR prevalence. Patients were sampled with replacement from the CPR and non-CPR subgroups in the specified ratio, while preserving the original trial size of 420 patients to ensure comparability. Univariable logistic regression was applied to each synthetic cohort and the process was repeated 1,000 times per scenario to account for random variation in sampling. The distribution of estimated treatment effects was then summarized by the mean OR and the. 2.5th and 97.5th percentiles of the bootstrap distribution, providing empirical CIs. This approach reflects how results might vary if the trial had enrolled different proportions of resuscitated patients.

## Results

**Figure 1:**
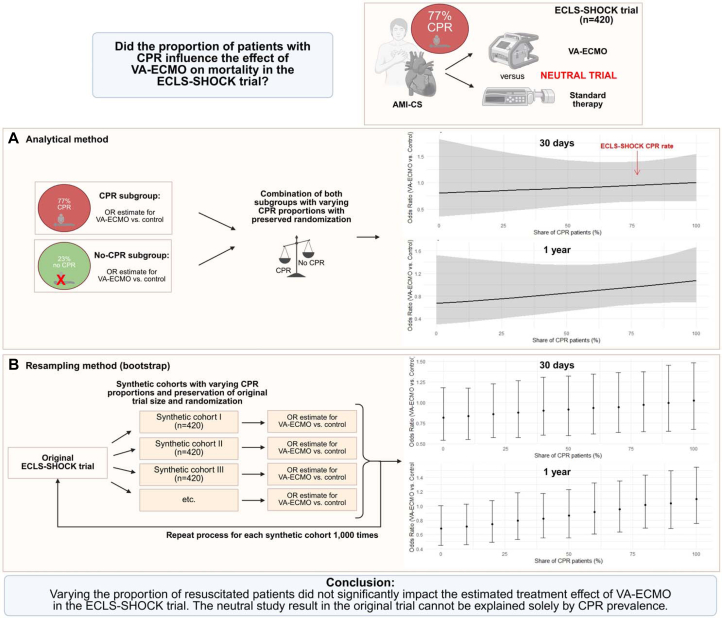
**Effect of Venoarterial Extracorporeal Membrane Oxygenation on Mortality Across Varying Cardiopulmonary Resuscitation Proportions** Illustration of 2 statistical approaches assessing whether the proportion of patients with preceding CPR influenced the effect of VA-ECMO on 30-day and 1-year mortality in the ECLS-SHOCK trial. (A) Analytical method: subgroup-specific ORs for CPR and non-CPR patients were mathematically combined across varying proportions of CPR prevalence. (B) Resampling method (bootstrap): synthetic trial cohorts of fixed sample size were repeatedly generated at different CPR proportions and ORs were estimated across 1,000 iterations. Both methods consistently demonstrated treatment neutrality of VA-ECMO across the full range of CPR prevalences. Created in BioRender and is licensed under https://creativecommons.org/licenses/by/4.0/. Desch, S. (2025) https://BioRender.com/v705w0n. AMI-CS = acute myocardial infarction complicated by cardiogenic shock; CPR = cardiopulmonary resuscitation; ECLS-SHOCK = Extracorporeal Life Support Infarct-Related Cardiogenic Shock; VA-ECMO = veno-arterial extracorporeal membrane oxygenation.

In patients with CPR, the OR for 30-day mortality with VA-ECMO vs control was 1.0 (95% CI: 0.65-1.55). In patients without CPR, the corresponding OR was 0.81 (95% CI: 0.36-1.82). Across both the analytic mixture and resampling approaches, the estimated treatment effects consistently hovered around neutrality, indicating no survival advantage of VA-ECMO over control. When the assumed proportion of resuscitated patients was lower, the estimated OR shifted toward potential benefit with VA-ECMO (OR: 0.7-0.8), but the CIs consistently spanned unity, precluding statistical significance. The resampling method showed narrower CIs (given larger synthetic cohorts of 420 patients each), yet the overall treatment effect again remained centered around unity across all CPR proportions. Analyses on 1-year mortality outcomes yielded similar results (Figure 1).

## Discussion

This subanalysis of the ECLS-SHOCK trial showed that the estimated treatment effect of VA-ECMO on 30-day and 1-year mortality remained neutral across the entire spectrum of CPR rates. Given that 77% of patients in the ECLS-SHOCK trial had undergone CPR, it has been widely argued that this case mix may have obscured a potential benefit of VA-ECMO.

By contrast, the DanGer Shock trial enrolled only 20% resuscitated patients (all of whom were noncomatose) and demonstrated a survival advantage of microaxial flow pumps at longer follow-up, fueling speculation that CPR accounted for the differing results. A prior analysis of predefined subgroups demonstrated no significant interaction between CPR status and clinical outcomes.^4^ Given the overlapping CIs in resuscitated and nonresuscitated patients, the absence of a statistically robust subgroup effects is underscored, thereby supporting that the neutral overall finding is unlikely to be explained by CPR prevalence alone.

Collectively, these findings reinforce the statistical robustness that the neutral result of ECLS-SHOCK cannot be attributed to resuscitation status alone. Other differences between the 2 trials, such as the devices themselves, stricter patient selection (eg, ST-segment myocardial infarction with left ventricular failure), and timing of initiation of mechanical circulatory support, and also the noticeable 12% mortality increase from day 30 days to 6 months in the control group in the DanGer Shock trial may explain some of the differences in outcome.^5^

Limitations include reliance on independent assumptions in the analytic mixture, resampling restricted to the original sample size, and no adjustment for CPR-specific prognostic factors. The analytic approach was underpowered to detect modest subgroup benefits, as reflected by wide CIs. Therefore, resampling with synthetic cohorts of 420 patients was performed showing narrowed CIs but still no significant effect. Thus, these findings remain hypothesis-generating and do not exclude type II error. The overlapping CIs in CPR and non-CPR subgroups underscore the absence of robust subgroup effects, supporting that the neutral outcome is unlikely to be explained by CPR prevalence alone. Nevertheless, consistency across 2 independent methods reinforces the robustness of the overall findings.

In summary, varying the proportion of resuscitated patients did not materially change the estimated treatment effect of VA-ECMO. These findings suggest that CPR prevalence alone is unlikely to explain the neutral result of ECLS-SHOCK; however, given the post hoc and theoretical nature of the analysis, these results should be interpreted as hypothesis-generating.

## Funding support and author disclosures

The authors have reported that they have no relationships relevant to the contents of this paper to disclose.
